# Parental Cognitions About Sleep Problems in Infants: A Systematic Review

**DOI:** 10.3389/fpsyt.2020.554221

**Published:** 2020-12-21

**Authors:** Susanne Knappe, Anna-Lisa Pfarr, Johanna Petzoldt, Samia Härtling, Julia Martini

**Affiliations:** ^1^Institute of Clinical Psychology and Psychotherapy, Technische Universität Dresden, Dresden, Germany; ^2^University of Meißen (FH) and Centre of Further Education, Meißen, Germany; ^3^Department of Psychiatry and Psychotherapy, Faculty of Medicine, Carl Gustav Carus University Hospital, Technische Universität Dresden, Dresden, Germany

**Keywords:** infant, child, sleep problem, parental, maternal, paternal cognition

## Abstract

**Introduction:** Parental cognitions may directly and indirectly contribute to infant sleep outcomes. This review provides a systematic up-to-date overview of the associations between parental cognitions and infant sleep problems with special emphasis on temporal relationships and the content of parental cognitions.

**Methods:** A systematic literature research in PubMed and Web of Science Core Collection sensu Liberati and PRISMA guidelines was carried out in March 2020 using the search terms (parent^*^ AND infant^*^ AND sleep^*^ problem^*^), including studies with correlational or control group designs investigating associations between parental cognitions and sleep problems in children aged 1–6 years.

**Results:** Twenty-three studies (published from 1985 to 2016) met inclusion criteria, of which 14 reported group differences or associations between parental sleep-related cognitions and child sleep outcomes. Nine papers additionally reported on the role of general parental child-related cognitions not directly pertaining to sleep. Findings from longitudinal studies suggest that parental cognitions often preceded child sleep problems. Cognitions pertaining to difficulties with limit-setting were especially prevalent in parents of poor sleepers and were positively associated with both subjective and objective measures of child sleep outcomes.

**Conclusions:** Parental cognitions appear to play a pivotal role for the development and maintenance of sleep problems in young children, arguing that parents' attitudes and beliefs regarding child sleep inadvertently prompts parental behavior toward adverse sleep in offspring. Associations are however based on maternal reports and small to moderate effect sizes. Thus, additional parental factors such as mental health or self-efficacy, as well as additional offspring factors including temperamental dispositions and regulatory abilities, require consideration in further studies.

## Introduction

Infant sleep patterns develop rapidly over the 1st year of life. During the 1st months of development, infant sleep is fractionated into numerous sessions that last for about 4 h throughout the day. After 6 months, sleep becomes more stable, with night sleep duration increasing and daytime sleep decreasing simultaneously (1, 2). By the age of 12 months, most infants no longer need to be fed at night and sleep patterns further consolidate, with longer periods of uninterrupted sleep and a smaller number and duration of nighttime awakenings (1, 3–6). Albeit the majority of infants develop stable sleeping behaviors and self-soothing abilities at 12 months of age (7), sleep continues to be fragmented and disrupted in some infants after this period (8). Hence, sleep issues in toddlerhood and childhood are a major parental concern in childrearing, a primary reason for the interruption of parental sleep, and a common subject of referrals to professional care (9–11). According to parents and child-care professionals, bedtime, or night waking problems are the most common infant sleep problems (8, 10, 12–14). Prevalence rates of infant sleep problems consistently range between 20 and 30 percent across studies in newborns, infants, and toddlers (12, 15, 16). When left untreated, they pose a substantial risk to persist (8) until preschool age (17), leading to a number of adverse outcomes in later child development such as behavioral problems or lower cognitive performance [(18–20)]. In addition, sleep problems interfere with parental mental and physical health (21, 22) and are associated with increased levels of parenting stress (23, 24).

Risk factors for sleep problems include child variables such as temperament (25) and genetic factors (26), parent-centered variables such as parental mental health (22, 27) and psychological functioning (28), as well as marital stability (29, 30), and parenting behaviors promoting the implementation/consolidation of stable bedtime routines (31–33) or parental involvement at nighttime (34) [see (35) for a recent overview]. Sadeh et al. (36) reviewed that parental behaviors such as interactions at bedtime, soothing strategies and limit-setting strategies were linked directly to infant sleep variables. In fact, active parental involvement at nighttime and excessive comforting have consistently been linked to sleep onset difficulties and night awakening problems in infants and young children (7, 34, 37–42). Soothing, holding or feeding until the child falls asleep, as well as parental presence at bedtime in general, may interfere with the child's development of self-soothing abilities and falling asleep independently. Following Sadeh et al., excessive parental concerns regarding the limitation of personal involvement at nighttime were associated with disrupted sleep in toddlers and infants. In contrast, minimal parental assistance and early encouragement of infants' autonomy were associated with more consolidated sleep (36). As parental cognitions drive parents' behaviors around infant sleep, a better understanding of the temporal relationships and contents of parental expectations, attitudes, concerns, and beliefs is warranted to inform targeted behavioral interventions for improving infants' sleep.

In sum, this review contributes to the field by an actualized literature overview, considering specific parental cognitions, and extending the infant age range up to preschoolers. The term cognition is understood as perceptions, attitudes and beliefs regarding their child's behavior (here: child's sleep) including concerns, worries, and fears about the child's sleep. In this sense, Sadeh et al. included four studies directly examining the link between cognitions (defined as perceptions, attitudes, expectations, and interpretations about infant sleep) and sleep (43–46), and recapitulate that parental cognitions that expressed difficulties with limit-setting were most strongly associated with problematic infant sleep behaviors, suggesting that together with parental health and feeding concerns, parental cognitions regarding limit-setting might be particularly important when dealing with sleep onset or night waking issues in young children. Since completion of their review 10 years ago (in 2010), additional studies have added to this empirical base. Furthermore, specific parental cognitions about infant sleep or bedtime situations [cf. (36)] should be broadened to more general cognitions on parental feeding and safety concerns as well as parental self-efficacy during bedtime procedure. These cognitions may remain relatively stable across a variety of parent-child interactions and thus affect child sleep quality even when not specifically related to sleep situations. As sleep problems have been shown to persist in older children such as preschoolers (8), we will also review research evidence pertaining to parental cognitions for sleep onset difficulties and night waking problems beyond infancy and toddlerhood, up to preschool age.

We systematically review associations of both specific and general parental child-related cognitions (expectations, attitudes, concerns, and beliefs) with sleep problems in toddlers and young children up to 6 years of age. Second, we expect parental cognitions regarding limit-setting and concerns about their child's well-being to be more strongly associated with sleep problems, as compared to other cognitions such as doubt about parenting competence. Based on previous findings on the direction of associations, we argue that parental cognitions in favor of limit-setting are associated with less sleep problems, whereas parental concerns are expected to be positively associated with sleep problems in children.

## Materials and Methods

A systematic literature review sensu Liberati et al. (47) and the PRISMA guidelines of the electronic databases PubMed and Web of Science Core Collection was undertaken. The same search algorithm was also applied for random search in Ebscohost databases (including APA PsycArticles, APA PsycInfo, PSYNDEX Literature with PSYNDEX tests). Publications were considered eligible if original articles were published in English or German before March 2020. Given that stable sleep patterns in infants typically emerge at the end of the 1st year of life (48), and to account for school entrance as a developmental milestone (49), only studies that assessed outcome variables in toddlers and preschool children between the age of 12 months and 6 years were included. For studies examining children of mixed ages, a sample mean between 8 months and 6 years, as well as statistically controls for age in all relevant data analyses was required. Papers were included when the association between parental cognitions and child sleep variables was examined using either a correlational or control group design.

### Variables Assessed

*Parental cognitions* were defined as any cognition pertaining to the (own) child, child behavior, interactions with the child and the relationship with the child, as well as cognitions regarding parenting and the own role as a parent. General cognitions refer to stable parental feeding and safety concerns as well as parental self-efficacy during, but not limited to, bedtime procedure. In contrast, specific cognitions pertain to specific infant sleep or bedtime situations. Studies assessing more general concepts not directly related to parenting and/or the child, for example overall self-efficacy in major depression, were excluded, as well as studies examining parental knowledge (rather than cognitions) on child sleep and healthy sleep practices. Studies mixing up cognitions and behaviors that were not measured independently, and therefore did not allow for separate interpretation of results, were also excluded [e.g., in case of the overall stress score on the Parenting Stress Index-Short Form [PSI-SF; (50)]].

*Child sleep problems* were defined by sleep onset difficulties including sleep onset latency, bedtime resistance, nocturnal awakening that require parental intervention [e.g., soothing strategies, reunions with the parent; [(48, 51–53)]. Across classificatory systems and guidelines for clinically diagnosing sleep disorders in toddlers and young children, there seems to be common ground across parents and child-care professionals regarding the adversity of infant difficulties to initiate or maintain sleep during the night, which are referred to as bedtime or night waking problems. We thus only included studies that assessed sleep onset latency or the presence, number and duration of nocturnal awakenings in children from 1 to 6 years. Sleep efficiency describes the amount of time a child is asleep at night in proportion to the actual time spent in bed, including nocturnal awakenings and sleep onset latency. It was therefore considered as reliable proxy to the aforementioned variables. Likewise, in clinical samples, primary diagnostic criteria were required to comprise sleep onset latency and nocturnal awakenings, in separation from other sleep problems such as nightmares, somnambulism or sleep disordered breathing.

### Search Strategy and Data Extraction

**Figure 1:**
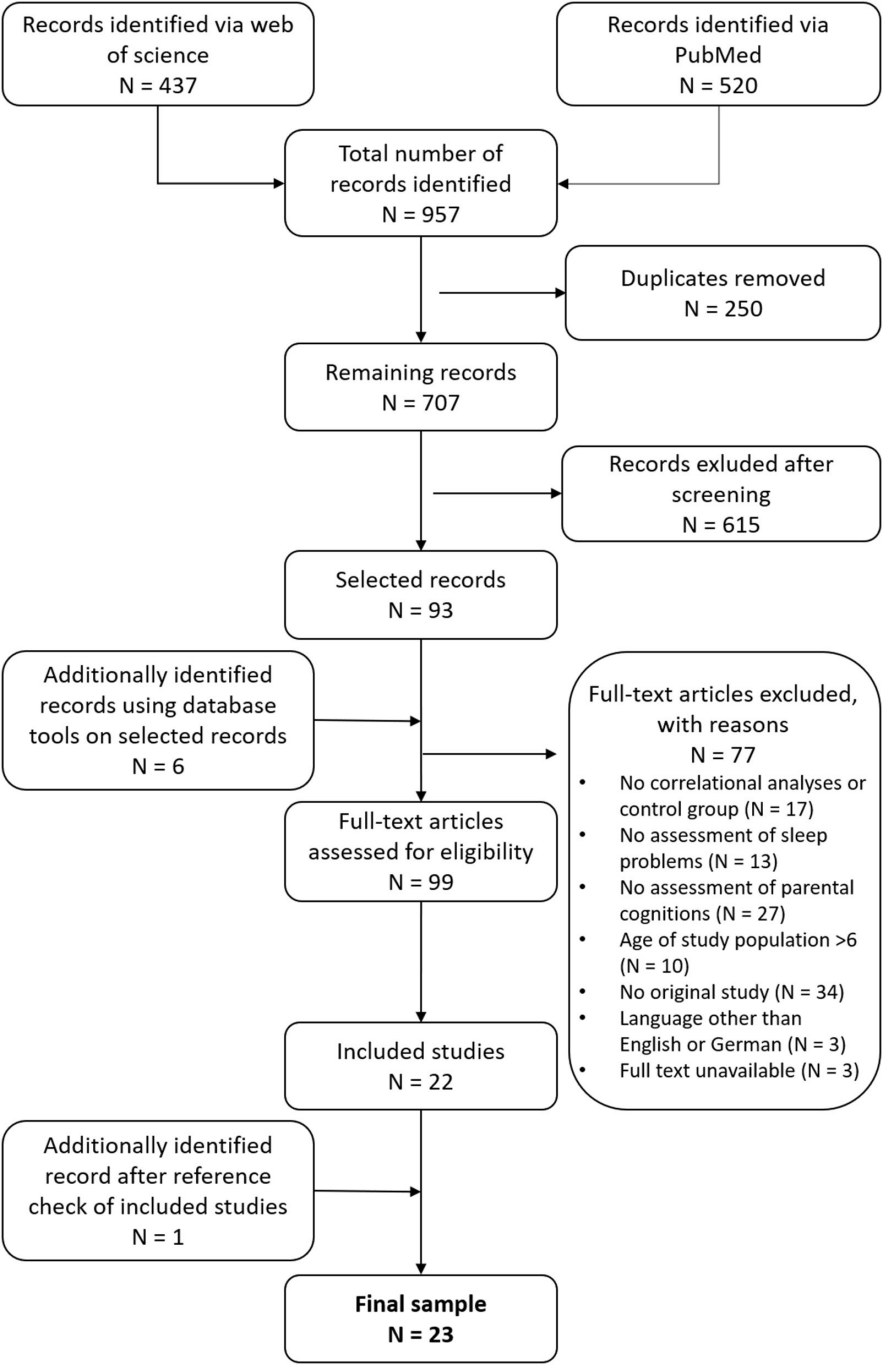
Flow chart of study selection.

*Identification of relevant papers* was based on a systematic literature research in PubMed and Web of Science Core Collection in March 2020 (Figure 1). The same search algorithm was also applied for random search in Ebscohost databases (including APA PsycArticles, APA PsycInfo, PSYNDEX Literature with PSYNDEX tests) without identifying additional publications for the review. Searches were conducted in accordance with the PICO approach (47) using the search terms: (parent^*^ AND infant^*^ AND sleep^*^ problem^*^), with key words referring to the targeted study population (infants), intervention or exposure (parenting), and control group and outcome (sleep problems). Screening of selected records and their reference lists, as well as Google Scholar and ResearchGate were used to identify publications not otherwise listed in data bases. Ninety-nine full-texts were assessed for eligibility, of which 77 were excluded. Hereupon, a full-text analysis was carried out on *N* = 23 original study papers. Data extraction using a fully standardized protocol (see Supplementary Table 1), as well as screenings and full-text analyses were performed by ALP, SK, and JM. Duplicates were removed manually. Studies were excluded when they did not meet inclusion criteria. Reasons for exclusion were lack of correlational analysis or control group design (*N* = 16 studies), lack of assessment on sleep problems (*N* = 13 studies), or parental cognitions (*N* = 27), study population older than 6 years (*N* = 10 studies), other than original study (*N* = 4 studies: book chapters, reviews, meta-analyses), language other than English or German (*N* = 3 studies), and full text unavailable (*N* = 3 studies).

*Extracted data* included general study information such as name of authors, year of publication, sample size, child age at sleep assessment, sample type (community/clinical) and study design, as well as study specific information on sleep variables and parental cognitions, correlation coefficients, *p*-values, effect sizes and directions of group effects and group comparisons. In acknowledgment of the cultural diversity in parental sleep practices and child sleep outcomes (54, 55), information about the cultural and ethnical background and socioeconomic status (SES) of study samples was also collected.

## Results

### Study Characteristics

The majority of original studies (15/23) were published in 2006 or later (range 1985–2016). All included papers were written in English. Sample sizes varied across studies, from four papers (studies no. 9, 11, 15, 17) reporting on *N* < 50 participants, to three national surveys (studies no. 16, 19, 22) examining up to *N* = 2,000 subjects. All studies were observational studies of which *n* = 17/23 included cross-sectional control-group and correlational designs, and *n* = 5 included a longitudinal design. One study [Morrel and Steele (43), study no. 3] had both a cross-sectional correlational design and a longitudinal approach. The majority of studies was based on community samples, while five studies (studies no. 4, 12, 14a, 14b, 17) reported on a clinical sample compared to a community sample. One study (no. 11) examined children clinically diagnosed with Williams Syndrome, while another study (no. 7) examined children of parents raised under communal sleeping arrangements, who were especially prone to problematic sleep-related cognitions. Most studies were based on Caucasian samples from Australia, Canada, the UK and the USA, with only one study from the USA reporting diversity in their sample (no. 13). Notably, a total of ten studies (studies no. 4, 6, 7, 10, 12, 14a, 14b, 18, 21, 23) were conducted in Israel, making this cultural group particularly well-represented in the body of evidence assembled here. Most families were indexed as having a middle-high SES, with only four studies assessing families of more diverse SES.

Most studies included infants up to 3 years, while three studies (studies no. 5, 8, 11) included children up to 5 years (referred to as “preschoolers”). One study (no. 10) examined children between 4 and 6 years of age.

Of the 23 included studies, *n* = 14 reported on sleep-related cognitions. Four of these studies were also reviewed by [Sadeh et al. (36)] (Supplementary Table 2). Meanwhile, nine studies assessed child-related cognitions not specifically referring to sleep (Supplementary Table 3). One study (no. 14a, 14b) reported on both sleep-related and child-related cognitions.

## Question 1: Associations of Specific and General Parental Child-Related Cognitions With Sleep Problems

### Results for Specific Sleep-Related Parental Cognitions

Fourteen studies reported on the association of sleep-related cognitions in parents with offspring sleep outcomes (Table 1). For child sleep outcomes, the number of nocturnal awakenings (Seven studies: 4, 6, 7, 8, 10, 11), average duration of nocturnal awakenings (Two studies: studies no. 4, 8), sleep onset latency (Two studies: studies no. 4, 7), and sleep efficiency (study no. 10) were considered (Table 1). Four studies (studies no. 1, 5, 9, 13) used composite scores of a combination of sleep variables. The other studies (studies no. 1-4, 12, 14a) reported sum scores for diverse outcome measures on infant sleep.

**Table 1:** Studies on sleep related cognitions in parents and offspring sleep.

	**Authors**	**Cognition type**	**Cognition measure**	**Sleep variables**	**Sleep measure**	**Statistical analysis**	**Significant findings and effect sizes**
1	Morrell (46)^a^	Limit-setting (L) Anger (A) Doubt (D) Feeding (F) Safety (S) Total Score (T)	MCISQ	Identification of SPG and CG according to SOL, number and duration of NA and times slept in parental bed per week [criteria after (56)] Composite score comprising SOL, number and duration of NA, total sleep time per night and weekly hours in parental bed	ISQ Richman's sleep diary	Between- group differences in MCISQ scales using Mann- Whitney *U*-tests (two-tailed) Pearson correlations between composite sleep score and MCISQ scales	L (*p* = 0.0002) A (*p* = 0.013) D (*p* = 0.0095) T (*p* = 0.017) (SPG > CG for all scales) L (*r* = 0.52; *p* < 0.001) A (*r* =0.39; *p* = 0.002) D (*r* = 0.37; *p* = 0.004) T (*r* = 0.48; *p* < 0.001)
2	Morrell and Cortina- Borja (38)^a^	Composite score comprising the subscales limit-setting, anger and doubt (CS)	MCISQ	Identification of SPG and CG according to SOL, number and duration of NA and times slept in parental bed per week [criteria after (56)]	ISQ	Between- group differences in MCISQ composite score using one way ANOVA with *post-hoc* comparisons of means (Tukey test)	CS (*p* < 0.001) (SPG > CG)
3	Morrell and Steele (43)	T1: Limit-setting (L) Anger (A) Doubt (D) T2: L&A composite score	MCISQ	Identification of SPG and CG at T1 according to SOL, number and duration of NA and times slept in parental bed per week [criteria after (56)] and according to maternal criteria at T2	ISQ (T1 and T2)	T1: (cross-sectional) Logistic regression analysis predicting group membership while controlling for child age T2: (longitudinal) Spearman correlations between L&A and continuous sleep problem group membership (member of SPG at T1 and T2)	T1: L (*R^2^* = 0.25; *p* < 0.000) A (*R^2^* = 0.11; *p* = 0.001) T2: L&A (^r^s = 0.528; *p* < 0.001)
4	Sadeh et al. (44)^b^	Limit-setting (L) Anger (A) Doubt (D) Feeding (F) Safety (S) Distress (DI) Limits (L-ISVIS) Temperament (TE)	PCISQ ISVIS	Number of NA, SOL, wakefulness (= total duration of NA during the night) SPG= clinical sample searching help at a child sleep laboratory CG= children who had never received professional help for sleep problems (significant differences between SPG and CG in number and duration of NA)	Actigraphy-based sleep monitoring Sleep diary (both administered for one week)	Pearson correlations between PCISQ and ISVIS cognition scales and sleep variables measured objectively and subjectively Between-group differences in MCISQ and ISVIS scales using MANCOVA	Wakefulness (obj.): D (*r* = −0.33; *p* < 0.001) Wakefulness (subj): L (*r* = 0.40; *p* < 0.001) A (*r* = 0.28; *p* < 0.05) TE (*r* = 0.23; *p* < 0.05) Number of NA (obj.): D (*r* = −0.22; *p* < 0.05) Number of NA (subj.): L (*r* = 0.28; *p* < 0.01) A (*r* = 0.24; *p* < 0.05) SOL (obj.): A (*r* = 0.21; *p* < 0.05) D (*r* = 0.30; *p* < 0.001) Between-group differences: L (*F* = 11.88; *p* < 0.001 L-ISVIS (*F* = 4.46; *p* < 0.05) (SPG > CG for both scales)
5	Johnson and McMahon (15)	Composite score comprising 14 items on limit-setting, anger and doubt (CS)	MCISQ	Composite score comprising SOL, number and duration of NA, time slept in parental bed and use of sleep aids	TCSQ	Correlational analysis between sleep and cognition composite scores	CS (*r* = 0.39; *p* < 0.01)
6	Tikotzky and Sadeh (39)	Distress (DI) Limits (L-ISVIS) (assessed during pregnancy, at 1, 6 and 12 months)	ISVIS	Number of NA	Actigraphy-based sleep monitoring Sleep diary (both administered four consecutive nights) BISQ	Pearson correlations between number of NA assessed by actigraphy, sleep diary and BISQ and ISVIS scales SEM-analysis (maximum likelihood method) cross legged effects of 6 months parental cognitions (D&L-ISVIS) on 12 months infant NA(subj. and obj.) after controlling for infant sleep at 6 months and vice versa	NA sleep diary: DI *p* (*r* = 0.22; *p* < 0.05) DI 6m (*r* = 0.25; *p* < 0.05) DI 12m (*r* = 0.28; *p* < 0.01) L p (*r* = −0.21; *p* < 0.05) L 1m (*r* = −0.26; *p* < 0.05) L 6m (*r* = −0.28; *p* < 0.01) L 12m (*r* = −0.27; *p* < 0.01) NA BISQ: DI 6m (*r =* 0.36; *p* < 0.001) DI 12m (*r =* 0.25; *p* < 0.05) L 1m (*r =* −0.23: *p* < 0.05) L 6m (*r =* −0.41; *p* < 0.001) L 12m (*r =* −0.27; *p* < 0.05) Cognitions 6m on sleep 12m (*P* = 0.46; *p* < 0.05) Sleep 6m on cognitions 12m (*P* = −0.27; *p* < 0.05)
7	Tikotzky et al. (45)^b^	Limit-setting (L) Anger (A) Doubt (D) Feeding (F) Safety (S) Distress (DI) Limits (L-ISVIS) Temperament (TE)	PCISQ ISVIS	Number of NA Number of NA and SOL	Sleep diary (administered five consecutive nights) BISQ	Pearson correlations between PCISQ and ISVIS cognition scales and number of NA and SOL	NA sleep diary: L (*r =* 0.21), F (*r =* 0.23), DI (*r =* 0.23) NA BCISQ: L (*r =* 0.18), D (*r =* 0.17), TE (*r =* 0.19) SOL BCISQ: F (*r =* 0.19), S (*r =* 0.19), L-ISVIS (*r =* −0.19) (*p* < 0.05 for all correlations)
8	Coulombe and Reid (57)	Limit-setting (L-NVS) Active comforting (AC) Reward (R) Punishment (P)	NVS	Number of NA per night and week (multiplied) Duration of average NA	ISQ- A	Pearson correlations between NVS-scales and number and duration of NA	Number of NA: L-NVS (*r =* −0.20; *p* < 0.01) AC (*r =* 0.30; *p* < 0.01) P (*r =* −0.18; *p* < 0.05) Duration of NA: L-NVS (*r =* −0.22; *p* < 0.01)
9	Teti and Crosby (58)	Worries about infant's physical and emotional needs (9 items of L&F) Helplessness and loss of control (3 items of A&D)	MCISQ	Night waking score containing information of average number and duration of NA	Infant sleep diary (administered seven consecutive nights)	Partial correlations between maternal cognitions and night waking score statistically controlling for child age	Worries (*r =* 0.36; *p* < 0.05)
10	Tikotzky and Shaashua (59)	Limit-setting (L) Distress (DI) Limit-setting (L-ISVIS) Limit-setting composite score (LSC) comprising transferred L-scores and L-ISVIS-scores (assessed at 12 months)	MCISQ ISVIS	Number of NA Sleep efficiency Number of NA Number of NA	Actigraphy-based sleep monitoring Sleep diary (both administered four consecutive nights) BCSQ	Pearson correlations between MCISQ and ISVIS scores at 12 months and subjective and objective sleep measures at 4 years Multiple regression analyses with LSC scores at 12 months as predictors of NA (obj.) and sleep efficiency (obj.) at 4 years	NA (obj.): L (*r = 0.3*6; *p* < 0.001) L-ISVIS (*r = –*0.28; *p* < 0.05) LSC (*r = –*0.35; *p* < 0.001) Sleep efficiency (obj.): L (*r = –*0.27; *p* < 0.05) LSC (*r =* 0.27; *p* < 0.05) LSC at 12 months on NA (obj.) at 4 years (β = –0.27; *p* < 0.05; explained variance =7%)
11	Axelsson et al. (60)	Distress (DI) Limits (L-ISVIS) Temperament (TE)	ISVIS	Number of NA	BISQ	Kendall's t correlations between NA and ISVIS- scales calculated separately for WS and TD group	Group (TD): DI [t_(12)_*=* 0.55; *p = I*15) L-ISVIS [t_(12)_ = –0.56; *p =* 0.013)
12	Golik et al. (61)^a^	Limit-setting (L) Anger (A) Doubt (D) Feeding (F) Safety (S) Total (T)	MCISQ	SPG= children diagnosed as having behavioral insomnia of childhood based on ICSD criteria CG = children never having received professional help for sleep problems		Between-group differences in MCISQ scales using independent *t*-tests or ANOVA	T (*p* < 0.01) A (*p* < 0.05) D (*p* < 0.05) F (*p* < 0.05) (SPG > CG for all scales)
13	Lemery- Chalfant et al. (62)^a^	Total (T)	MCISQ	Composite sleep dysregulation score comprising sleep onset difficulties, night waking and need of parental assistance in falling asleep (five items)	ITSEA	Multilevel processing to test process models	T significantly predicted infant sleep dysregulation score
14a	Sadeh et al. (63)^b^	Distress (DI)	ISVIS	SPG = couples who sought help for their infant night waking problems CG1 = couples with no reported infant sleep problems CG2 = childless married		General linear mixed models based on the Proc Mixed procedure ^c^ with group membership as independent variable and *post-hoc* comparisons of means	DI (*F* = 6.93; *p* < 0.005) SPG > CG2 (*t* = 3.46; *p* < 0.0005)

*Papers are listed by publication year and then alphabetically. For the sake of parsimony, only significant findings are reported in the last column*.

*MCISQ, Maternal Cognitions about Infant Sleep Questionnaire; SPG, sleep problem group; CG, control group; SOL, sleep onset latency; NA, nocturnal awakenings; ISQ, Infant Sleep Questionnaire; T1, time one; T2, time two; PCISQ, Parental Cognitions about Infant Sleep Questionnaire; ISVIS, Infant Sleep Vignettes Interpretations Scale; obj., objectively; subj., subjectively; TCSQ, Tayside Children's Sleep Questionnaire; BISQ, Brief Infant Sleep Questionnaire; NVS, Night Waking Vignettes Scale; ISQ-A, Infant Sleep Questionnaire adapted for use by parents of preschool-aged children; BCSQ, Brief Child Sleep Questionnaire; ITSEA, Infant Toddler Social and Emotional Assessment*.

a *Studies solely reporting p-values, without giving information about effect sizes or reporting neither p-values nor effect sizes*.

b *Studies assessing cognitions of fathers in addition to those of mothers. Significant findings are presented for both mothers and fathers. In case of separate analyses and significant outcomes for both genders, only those of the mother were reported in order to maintain a clear and decided structure of tables*.

c *After (64)*.

### Results for General Child-Related Parental Cognitions

Significant associations between parental cognitions not specifically pertaining to child sleep and child sleep variables were expected. These findings are summarized in Table 2.

**Table 2:** Studies on child-related cognitions not referring to sleep.

	**Authors**	**Cognition type**	**Cognitions measure**	**Sleep variable**	**Sleep measure**	**Statistical analysis**	**Significant findings and effect sizes**
15	Lozoff et al. (65)	Categorical variable: Maternal accepting attitude toward the child yes/no (MAA)	Interview ratings using Ainsworth's one-to-nine-point scale ranging from one highly rejecting to nine highly accepting [interrater reliability = 85%; (54)]	Identification of SPG and CG according to presence/absence of night waking problems or bedtime struggles occurring at least three times per week during the months prior to the interview	Standardized interview carried out by two independent coder- raters at the pediatric facility [mean agreement per item =95%; (54)]	Between-group differences in MAA using Fisher exact test Discriminant analysis	MAA (*p* = 0.006) (SPG < CG) MAA contributed to discriminating SPG from CG at a significant level (F=1.11) as part of a model comprising five variables [*F*_(5, 26)_ = 9.39; *p* = 0.0001]
16	Scott and Richards (66)	Attitude toward motherhood (ATM) (“very negative/ unhappy” through “generally negative,” “partially negative,” “philosophical acceptance” (i.e., “it's all part of childhood”) to “neutral,” “generally positive,” and “very positive/ excellent”) Perception of baby as dominating (Baby does not dominate my life at all-totally dominates my life)	Ratings on 7-point scale using information from different sections of a questionnaire containing closed and open questions with spaces left open for the mothers to add their comments (57% of the questionnaires could be satisfactorily coded for this variable) 7-point dominating scale in questionnaire	Identification of SPG and CG according to number of NA per week (SPG> five nights; CG < five nights)	Night waking scale in questionnaire containing closed and open questions with spaces left open for the mothers to add their comments	Between-group differences in ATM using independent *t*-tests	ATM (*p* < 0.001) (SPG < CG) Dominating scale: yes (*t* = 5.69; *p* < 0.001) (SPG>CG)
17	Benoit et al. (67)	Classification of the mother's overall perceptions of and relationship with her infant (balanced, disengaged or distorted)	Ratings of recorded information obtained in the Working Model of the Child Interview using eight anchored, 5-point rating scales (Interrater agreement= 0.53% in this study)	Identification of SPG and CG according to SOL, number and duration of NA and times slept in parental bed per week [criteria after (56)]		Between-group differences in classification type using Pearson's chi-squared test *(x^2^)* for categorical data	Balanced (*x*^2^ = 7.51; df = 2; *P* = 0.02; Cramer V = 0.45) (SPG < CG)
18	Scher and Blumberg (68)	Categorical variable: Maternal Separation Anxiety (MSA) (low, average, high levels of separation concerns) Categorical variable: Maternal orientation toward the infant (MO) (Facilitator, Regulator, Reciprocator and Bipolar) (assessed at 6 months, respectively)	ESI (administered following a brief separation event) FRQ	Dichotomous variable: “Wakers” (=Children waking up at least once every night; 53% of included children) vs. “Non-wakers” (47% of children) SOL in minutes Average number of NA per night	Sleep Questionnaire	Group differences in MSA between wakers and non wakers using Pearson's chi- squared test (*x*^2^) for categorical data Group differences in SOL and NA between MO- categories using ANOVA (group differences in NA were only significant in firstborns, *n* = 32)	MSA (*x*^2^ = 5.5; *P* < 0.05) (SPG > CG) SOL (*F* = 2.88; *p* < 0.05) (Bipolar> Reciprocator) NA (*F* = 2.95; *p* < 0.05) (Facilitators < Reciprocators and Bipolars < Regulators)
19	Touchette et al. (69)	Maternal feelings of self efficacy (SE) and overprotectiveness (OP)	PPBS	Identification of SPG and CG according to presence of NA (SPG= sleeping more than 6 consecutive hours per night; CG = sleeping less than 6 consecutive hours per night)	SAQM	Probability of group membership as predicted by maternal cognitions using a Poisson regression model with estimation of risk ratios with a 95% CI	None
20	Simard et al. (25)	Maternal feelings of SE and sense of parental impact (SPI) (assessed at 5, 17, and 29 months)	SAQM	Dichotomous variable: SOL > 15 min vs. SOL <15 min	SAQM	Group differences in maternal cognition variables between SOL > 15 min and SOL <15 at T1, T2, and T3 using *t*-tests	T1, T2 and T3: SE at 5, 17 and 29 months (SPG < CG) SPI at 5 and 17 months (SPG < CG)
21	Millikovsky- Ayalon et al. (70)^a^	Perception of parental role as restrictive (RR) (e.g. “Feel trapped by parenting responsibilities”)	Role restriction scale (four items) from the PSI-SF	Identification of SPG and CG according to number of NA per night/week [criteria after (56)]	Screening Questionnaire based on (56) criteria	Between-group differences in RR using independent *t*-tests	RR (*p* < 0.05; effect size = 0.49) (SPG > CG)
22	Zaidman-Zait and Hall (71)	Maternal SE, SPI, and OP (assessed at 5, 17, and 29 months)	PACOTIS	Categorical variable according to presence and average duration of NA: no waking waking <20 min waking > 20 min	SAQM	Group differences in parental cognitions between three NA categories using repeated ANOVAs and *post hoc* tests	Cognitions at 29 months: SE (*F* = 13.08; *p* < 0.001) (3 <2 <1); SPI (*F* = 5.57; *p* < 0.01) (3 <2 <1); OP (*F*= 9.45; *p* < 0.001) (1 <2 <3) Cognitions at 5 and 17 months: SE (3 <2 <1); SPI (3 <2 <1); OP (1 <2 <3) Main effects of wake group across all time periods: SE (*F* = 15.24; *p* < 0.001) (3 <2 <1); SPI (*F* = 7.52; *p* < 0.01) (3 <2 <1); OP (*F* = 8.94; *p* < 0.001) (1 <2 <3)
14b	Sadeh et al. (63)^a^	Distress level parents attributed to infant crying sounds (distress interpretation of infant crying)	RICAS^b^ procedure including ratings on six 10- point Likert-like scales after each presentation of a total of ten 30s audio-recordings of infant crying in varying intensities and tones	SPG =couples who sought help for their infant night-wakings problems CG1= couples with no reported infant sleep problems CG2=childless married couples		General linear mixed models based on the Proc Mixed procedure^c^ with group membership as independent variable and *post-hoc* tests	Significant group fixed main effects: Distress (*F* = 3.20; *p* < 0.05) SPG > CG1 (*t* = 2.19; *p* < 0.05)
24	Tikotzky (72)	Impaired Bonding (=the mother's feelings toward her infant and perceptions of her emotional relationship with the infant) Rejection and anger toward the infant Anxiety about care Acceptance/Tolerance Parental competence	PBQ MPAQ	Average number of times mother woke at night to attend to her infant = average number of maternal NA per night that were due to the infant	Sleep diary (administered five consecutive nights)	Pearson correlations between maternal cognitions and number of NA	Impaired Bonding (*r* = 0.33; *p* < 0.01) Rejection (*r* = 0.48; *p* < 0.001) Anxiety (*r* = 0.33; *p* < 0.01) Acceptance (*r* = −0.29; *p* < 0.05)

*Papers are listed by publication year and then alphabetically. For the sake of parsimony, only significant results are reported in the last column*.

*MAA, maternal accepting attitude; SPG, sleep problem group; CG, control group; ATM, attitude toward motherhood; NA, nocturnal awakenings; SOL, sleep onset latency; MSA, maternal separation anxiety; ESI, Emotional State Index; MO, maternal orientation toward the infant; FRQ, Facilitator-Regulator Questionnaire; PPBS, Parental Perceptions and Behaviors Scale; SE, self efficacy; OP, overprotectiveness; SAQM, Self-Administered Questionnaire for the Mother; SPI, sense of parental impact; RR, perception of the parental role as restrictive; PSI-SF, Parental Stress Index-Short Form; PACOTIS, Parental Cognitions and Conduct toward the Infant Scale; PBQ, Postpartum Bonding Questionnaire; MPAQ, Maternal Postnatal Attachment Questionnaire*.

a *Studies assessing cognitions of fathers in addition to those of mothers. Significant findings are presented for both mothers and fathers. In case of separate analyses and significant outcomes for both genders, only those of the mother were reported in order to maintain a clear and decided structure of tables*.

b *After (73)*.

c *After (64)*.

As shown in Table 2, seven studies (studies no. 14b, 15-17, 19, 21, 24) reported findings based on cross-sectional case control or control group designs, while three studies (studies no. 18, 20, 22) reported findings based on longitudinal (i.e., prospective cohort-designs). With regard to offspring sleep variables, most studies compared sleep problems with a control group, or used a control-group design. Others created categorical variables, however, categorization criteria for the sleep problem group and control group varied across studies. One paper (no. 14b) compared a group of infants receiving professional help for their sleep problems to two control groups, another two papers (studies no. 17, 21) identified sleep problem groups according to Richman's criteria (1981). Three studies (studies no. 15, 18, 22) classified infants with regard to the presence/absence of night waking problems or bedtime resistance as well as the number of nocturnal awakenings per week or sleep duration of <6 consecutive hours per night (no. 19). Sleep variables further comprised the number and duration of nocturnal awakenings and sleep onset latency in minutes for the purpose of group categorization (studies no. 18, 20, 22).

Overall, 10 studies (studies no. 14b, 15-22, 24) reported on eight distinct cognitions (indicated in italics) and their associations with child sleep problems. One study (no. 19) however, did not find an association between maternal self-efficacy and overprotectiveness with nocturnal awakenings. Three studies (studies no. 15, 16, 17) found higher levels of *maternal acceptance* and more *positive feelings and perceptions of the child* in controls than in sleep problem groups. Of note, maternal disengagement and distortion were unrelated to sleep problems in offspring (17). One study (24) also reported significant correlations between infant nocturnal awakenings and *impaired bonding, parental anger and rejection toward the infant*, as well as accepting parental attitudes toward the child. Two studies (16, 21) observed that parents of infants with sleep problems felt more *restricted by their parenting duties* and perceived their *child to dominate their lives* more strongly than parents of controls. Evidence pertaining to *parental self-efficacy and sense of parental impact* was based on two longitudinal studies (20, 22), and was not observed in cross-sectional studies (19, 24). Simard et al. [(25); study no. 20] found that parents of children with a sleep onset latency longer than 15 min at the ages of 50 months, 5 and 6 years felt less self-efficient as a parent when the child was at the ages of 5, 17, and 29 months. These parents also had a lower sense of parental impact when the child was aged 5 and 17 months. However, these associations attenuated to non-significance when analyses were adjusted for maternal depression. Similarly, self-efficacy and sense of parental impact measured at ages 5, 17, and 29 months were significantly lower in parents of infants with nocturnal awakenings longer than 20 min at the age of 29 months (22), when compared to nocturnal awakenings of <20 min or control groups. Higher levels of *maternal overprotection* at the ages of 5, 17, and 29 months predicted child sleep problem group-membership at 29 months, but only in a prospective study (22; but not in 19).

Scher and Blumberg [no. 18 (68)] examined whether maternal separation anxiety at 6 months was linked to the infant being a “waker” at the age of 12 months, and whether the number of nocturnal awakenings and child sleep onset latency at 12 months differed according to maternal regulation beliefs. Their results showed that higher *levels of separation-related concerns* were reported in mothers of infants who woke up at least once per night. Moreover, mothers classified as “Bipolars” more frequently had infants with longer sleep onset latency at 12 months, compared to other groups. Also, nocturnal awakening was reported most often in infants of mothers with dysfunctional regulation beliefs (pertaining to co-regulation and facilitation) as compared to mothers with more functional beliefs (i.e., Regulators and Facilitators). Finally, Sadeh et al. [(63), study no. 14b] found that parents of infants with sleep problems *attributed more distress to infant crying sounds* compared to parents of infants without sleep problems, but not compared to married couples without children.

## Question 2: Parental Cognitions Pertaining to Limit-Setting and Parental Concerns

Evidence on the particular role of limit-setting was found in nine studies (Table 1; 17 correlations; studies no. 1, 3, 4, 6, 7, 8, 10, 11, 12). As expected, cognitions on the importance of limit-setting were negatively correlated with problematic sleep behaviors, while difficulties with limit-setting were positively associated with sleep problems. Three studies compared a group of participants with sleep problems to a control group (studies no. 1, 3, 4). One of these studies (no. 12) did not reveal any differences in limit-setting between the groups (albeit differences were observed for doubts about managing infant sleep, and anger at infants' demands around). The remaining two studies (studies no. 1, 4) found that higher levels of unfavorable cognitions indicated difficulties with limit-setting in parents of poor sleepers. However, couples without children (i.e., hypothetical parents of poor sleepers) actually emphasized the importance of limit-setting more than parents of control infants (studies no. 4, 14a). Cognitions regarding limit-setting were more often reported in the sleep problem group (no. 3). Cognitions on limit-setting at 12 months also predicted the number of nocturnal awakenings at age 4 (no. 19). For one study (no. 12), no group differences emerged, and correlations were also not significant in a subsample of children clinically diagnosed for Williams Syndrome compared to significant findings in the comparison group (no. 11).

For parental concerns, as measured by the Maternal Cognitions about Infant Sleep Questionnaire (MCISQ) feeding and safety scales (studies no. 1, 4, 7, 12), only one out of four studies reported small effects, thereby linking feeding concerns to more nocturnal awakenings in community children, as well as to longer sleep onset latency (no. 7). Similarly, (61) (no. 12) found a greater level of parental feeding concerns in a selected sample of infants with sleep problems, as compared to control parents. Concerns about the infant being scared or distressed at night, combined with the urge to actively sooth and comfort the infant were examined in seven studies (studies no. 4, 6, 7, 8, 10, 11, 14a) of which five (studies no. 6, 7, 8, 11, 14a) reported positive associations with subjective measures of nocturnal awakenings. For three correlations, parental distress had been assessed up to 4 years before the report on child sleep outcome at 12 months (no. 6). Three other studies did not find associations with subjective or objective measures of child sleep (studies no. 4, 10, 11), however, in the Williams Syndrome subsample [(60); study no. 11] parental distress was associated with child sleep in the comparison group.

For cognitions expressing anger toward the infant and doubt about individual parenting competence (studies no. 1, 3, 4, 7, 9, 12), four studies (studies no. 1, 2, 3, 5) reported significant findings for composite scores on problematic sleep behaviors, as well as for the subjective number and duration of nocturnal awakenings and objective measures for sleep onset latency. Likewise, parents of infants with sleep problems scored higher on anger scales than parents of unaffected controls (studies no. 1, 3, 12). Parental doubts about their competencies were associated with objective (but not with subjective) sleep measures in one study (no. 4). Parental competency doubts were also positively associated with sleep onset latency and sleep problem composite scores, but negatively associated with the number and duration of nocturnal awakenings. These associations were weak to moderate. With regard to between-group differences, two studies reported more doubts in parents of infants with sleep problems (studies no. 1, 12). In contrast, one study found anger and doubt unrelated to nocturnal awakenings in infants (no. 9).

Apart from evidence for specific cognition scales, few studies reported on the common impact of parental cognitions on child sleep outcomes. For example, Morrell and Steele [(43); study no. 3] found that the combination of anger and cognitions on problems with limit-setting significantly predicted continuous sleep problem group- membership in toddlers. Similarly, a composite score of limit-setting, anger, and doubt was found to be significantly higher among parents of toddlers with sleep problems (no. 2), as well as being moderately associated with sleep problems in preschool children (no. 5). In a study by Teti and Crosby [(58); study no. 9], the combination of parental limit-setting and feeding concerns (but not helplessness and loss of control) was moderately associated with infant night waking. Finally, MCISQ total scores comprising all subscales of limit setting, anger, doubt, feeding and safety were significantly elevated in parents of children with sleep problems, compared to parents of controls (studies no. 1, 12). These scores were also positively correlated with child sleep dysregulation at 12 months (no. 13).

## Discussion

This systematic review aims to provide a comprehensive and up-to-date overview on the role of parental cognitions for sleep onset difficulties and night waking problems in toddlers and young children up to the age of six. A total of 23 studies were reviewed and findings are in line with a previous meta-analysis (36). Our review revealed associations for both general and sleep-related cognitions in parents, indicating that parental cognitions do not need to specifically focus on the child's sleep in order to be associated with child sleep problems. Moreover, parental cognitions expressing difficulties with limit-setting and concerns about the child's well-being were more strongly associated with child sleep problems than other parental cognitions such as doubt about parenting competence.

The majority of studies reported correlations between sleep onset difficulties and night waking problems. Three studies reported higher levels of problematic cognitions in parents of infants with sleep problems, compared to parents of infants without sleep problems. Effect sizes of correlations and between-group analyses were mostly in the weak to moderate range, indicating a moderate but stable effect of parental cognitions in child sleep. This is in line with studies of parental cognitions in other domains of child development such as feeding problems (74) and externalizing behavior (75). Only a few studies reported strong effects (studies no. 1, 3, 11). Associations were also observed in three prospective studies (studies no. 3, 6, 10), indicating that problematic parental cognitions may not only support the persistence of sleep problems, but also promote the development of fragmented sleep patterns in infants. In particular, limit-setting emerged as a substantial correlate or even predictor for sleep problems in infants. Results from longitudinal studies (no. 9) showed that toddlers' nocturnal awakenings at 12 months were associated with problematic parental cognitions dating back as far as pregnancy. Thus, problematic parental cognitions may arise in response to child behavior, as well as shaping child development from the very beginning of infancy, implying the existence of a mutual relationship. This is in line with longitudinal studies on parental cognitions promoting child behavior [study no. 21; (7, 36)], though not necessarily vice versa [i.e., (75, 76)].

There was similar evidence for the role of more general child-related cognitions for child sleep problems. Indeed, most of the eight general cognitions we reviewed were similar to the specific sleep-related cognitions in parents, with comparable effect sizes, suggesting that adverse parental cognitions at nighttime may actually reflect more general problematic parenting beliefs or attitudes. For example, maternal acceptance, which was higher in parents of good sleepers, might reflect the same or a similar construct such as anger, albeit with inverse scale orientation. In line with these considerations, one study (no. 24) reported significant associations between anger and rejection toward the child with the number and duration of child nocturnal awakening. Another study (no.m 14b), assessing parental distress attribution to infant crying sounds found positive associations with infant night waking, suggesting that parents of children with sleep problems interpret their infants' crying at nighttime as a sign of distress, leading to more anguish to their child's crying at any given time or situation. Longitudinal studies further argued for the predictive role of parental concerns, attitudes and beliefs to affect (promote) sleep onset and night waking problems in toddlers and young children. In turn, positive cognitions such as parental self-efficacy or accepting attitudes toward the infant may serve as protective factors for infants at risk for problematic sleep behaviors. Further, parental attitudes and beliefs that are *supportive* of limit-setting might serve as protective factors for infants at risk of developing sleep problems.

The review also points to some differences between cognitions, as the majority of studies reported on cognitions pertaining to limit-setting (as compared to other cognitions), with cross-sectional as well as longitudinal evidence of associations with all sleep variables in both toddlers and preschoolers. Cognitions pertaining to limit-setting may affect a variety of different sleep outcomes in infants, and have been linked to child sleep more often than other cognitions. This finding is in line with studies highlighting the importance of limit-setting practices and behaviors when trying to overcome sleep problems in children (7, 34, 36, 37, 41, 42), suggesting that limit-setting cognitions and behaviors are closely interrelated in parents. This result further stresses the importance of altering limit-setting cognitions when dealing with child sleep problems. On the other hand, there were no studies examining more general parental difficulties with limit-setting in contexts other than at nighttime, a subject that may be of interest for future research.

It also remains to be discussed to which degree general cognitions related to sleep problems shape parental cognitions and behaviors in other contexts. Intuitively, problematic parental cognitions regarding to some child behaviors may also extend and generalize to child behaviors in other contexts (cf. study no. 12). However, parental feeding and safety concerns were only weakly associated with child sleep problems and reported on by a smaller number of studies than other cognitions such as doubt about the own competence as a parent.

## Limitations

Findings of this review suggest that parental cognitions pertaining to limit-setting are more frequently involved in child sleep problems and prove more problematic for sleep outcomes than other cognitions. However, this systematic review does not qualify to determine effect sizes. Effect sizes of correlations and between-group analyses were mostly in the weak to moderate range. We did not perform a meta-analysis due to the considerable variability in sampling strategies, study designs and measurements of the majority of included studies. In particular, the reviewed studies included assessment instruments that do not clearly disentangle cognitive, emotional and behavioral outcomes (e.g., differentiation between parental cognitions, emotional responses, and actual parenting behaviors such as limit-setting). Also, the search terms sleep^*^ problem^*^ were included in this review, since this is an frequently used umbrella term in this multifaceted field of research. However, searching for the term “sleep” only would likely lead to more broader outcomes related to infant sleep. Adding the term “problem” denotes to a negative (deficit-focused) perspective on infant sleep; a maybe weaker (also less clinical-oriented) term could be “difficulties;” an even neutral term could be “behavior.” Since we defined sleep problems in line with current classification systems (see Methods), we aimed to capture clinically relevant (i.e., persisting and impairing) sleep behaviors in offspring, anticipating not to neglect significant contributions to this field of research. In addition, we also reviewed research evidence pertaining to parental cognitions for sleep onset difficulties and night waking problems beyond infancy and toddlerhood, up to preschool age.

Moreover, as there was no objective assessment of the quality of studies, more frequent and stronger effects for some cognitions might be due to publication bias or methodological differences across studies. Thus, we did not introduce an objective assessment of the quality of included studies such as systematically comparing information like number of participants, validity and reliability of measures or adjustment for confounding variables such as age of parents, infant sex, or time and place of sleep assessment. In particular, inter-parental differences in cognitions were not reported, nor was maternal depression, which is likely to be linked to parental cognitions (58, 61, 72) as well as child sleep problems [e.g., (77)]. Although many studies claim to also examine the mediating effects of parental cognitions on effective parental behavior toward child sleep variables (39, 57–59, 78), detailed inspection of these analyses was beyond the scope of this paper. However, as disentangling these associations is of great interest for clinical practice and intervention programs, subsequent reviews on parental cognitions should focus on mediation effects. Conclusions are also limited by the sample size (i.e., 23 studies included) and to predominantly white and Caucasian families of middle-upper SES, limiting generalizability to other cultural and socioeconomic backgrounds. Few studies included preschoolers, albeit the link between parental cognitions and child sleep still exists at school age and during adolescence (79–81). Similar to other reviews, this review is vulnerable to publication bias as only published studies and those to which we had access, were included. The majority of papers reported on maternal cognitions. Only four papers also included paternal cognitions, albeit differences between mothers and fathers were negligible.

## Conclusion

Problematic parental cognitions pertaining to child sleep or other realms of child development may contribute to the development of bedtime problems and fragmented sleep patterns in young children. Moreover, negative parental cognitions toward parenting or child behavior may extend to a variety of different interaction contexts and parenting situations, thus affecting parental behavior and child development in more than one context. Finally, results imply that a large part of problematic cognitions in child sleep pertains to parental difficulties with limiting their involvement at nighttime. Problematic cognitions included troubles setting limits as well as concerns that infants might experience distress upon awakening at night, inferring that parents should directly help and sooth their infant. Though findings are based on small associations und may be considered preliminary in particular for maternal cognitions, they however suggest including parental cognitions for interventions on offspring sleep, for example targeting limit setting in CBT-based interventions including psychoeducation and cognitive techniques. Since child sleep is predictive for later (cognitive) development, early interventions targeting specific sleep-related as well as general parental cognitions are likely to modify adverse parent-child-interactions and developmental trajectories.

## Data Availability Statement

The raw data supporting the conclusions of this article will be made available by the authors, without undue reservation.

## Author Contributions

SK prepared the manuscript. ALP, SK, and JM carried out the systematic literature research. Analyses and interpretation of the data by SK, ALP, JM, JP, and SH. ALP, JP, and JM helped to draft the manuscript. JP, JM, and SH critically revised the manuscript for important intellectual content. All authors contributed to the article and approved the submitted version.

## Conflict of Interest

The authors declare that the research was conducted in the absence of any commercial or financial relationships that could be construed as a potential conflict of interest.

## Supplementary Material

Supplementary Table 1 Checklist of items to include when reporting a systematic review or meta-analysis.

Supplementary Table 2 Sleep-related cognitions—study sample and design characteristics.

Supplementary Table 3 Child-related cognitions (not referring to sleep)—study sample and design characteristics.
