# Supplementary material for: Parental Cognitions About Sleep Problems in Infants: A Systematic Review
Source: Front Psychiatry. 2020 Dec 21;11:554221. doi: 10.3389/fpsyt.2020.554221 (PMC7779594; doi:10.3389/fpsyt.2020.554221)
Supplement: Supplementary Table 2 — Sleep-related cognitions—study sample and design characteristics. [file Table_2.pdf]

Supplemental Table 2: Sleep-related cognitions - Study sample and design characteristics

study no.	Authors	Publication year	Sample size	Child age at sleep assessment	Sample type and background information	Study design
1	Morrell	1999	*N* (Total) = 149	12-16 months	Community sample	Cross-sectional
			N (SPG) = 26	(mean= 13.72)	(predominantly white; English; middle-upper SES)	Control group design
			N (CG) = 123			
			N (SG) = 59			
2	Morrell & Cortina-Borja	2002	*N* (Total) = 99	12-19 months	Community sample	Cross-sectional
			N (SPG) = 40	(mean= 13.77)	(predominantly white; English; middle-upper SES)	Control group design
			N (CG) = 59			
3	Morrell & Steele	2003	*N* (Total) = 100	T1: 14-16 months	Community sample	Cross-sectional and longitudinal
			N (SPG;T1) = 40	(mean SPG= 15; mean CG= 15.3; significant difference)	(predominantly white; English; middle-upper SES)	Control group design
			N (CG;T1) = 60	T2: 26-28 months		
			N (SPG T1 & T2) = 25			
			N (SPG T1/not T2) = 15			
4	Sadeh et al.	2007	*N* (Total) = 96	4 - 29 months	Clinical sample and community sample control group (Israeli; middle SES)	Cross-sectional
			N (SPG) = 48	(mean SPG= 13.08; CG=		Case-control study
			N (CG) = 48	15.05; no significant difference)		
5	Johnson & McMahon	2008	*N* (Total) = 110	2 - 5 years	Community sample	Cross-sectional
				(mean= 3.81)	(predominantly Caucasian; Australian)	Correlational design
6	Tikotzky & Sadeh	2009	*N* (Total) = 85	12 months	Community sample	Longitudinal
					(Israeli; middle-upper SES)	Prospective cohort study

7	Tikotzky et al.	2010	*N* (Total) = 141	4.5-30 months (mean= 15.4)	Community sample (Israeli; middle-upper SES; 42 families with parents raised under communal sleeping arrangements in Israeli kibbutzim)	Cross-sectional correlational design
8	Coulombe & Reid	2012	*N* (Total) = 203	2 - 5 years<br>(mean= 3.4)	Community sample<br>(90% Caucasian; Canadian; diverse SES)	Cross-sectional<br>Correlational design
9	Teti & Crosby	2012	*N* (Total) = 45	1 - 24 months<br>(mean= 9.27)	Community sample<br>(91% white; US-American;<br>diverse SES)	Cross-sectional<br>Correlational design
10	Tikotzky & Shaashua	2012	*N* (Total) = 71	3.75 - 4.5 years<br>(mean= 4.15)	Community sample<br>(Israeli; middle-upper SES)	Longitudinal<br>Prospective cohort study
11	Axelsson, Hill, Sadeh & Dimitriou	2013	*N* (Total) = 28<br>*N* (WS) = 14<br>*N* (TD) = 14	18.03 – 47.47 months<br>(mean WS= 31.67; mean TD= 31.55; no significant difference)	Community sample<br>(WS-group: children with Williams Syndrome<br>TD-group: typically developing matched controls)	Cross-sectional<br>Correlational design
12	Golik et al.	2013	*N* (Total) = 201<br>*N* (SPG) = 31<br>*N* (CG) = 170	6- 36 months<br>(mean SPG= 13.4; CG= 16.5;<br>no significant difference)	Clinical sample and community sample control group (Israeli)	Cross-sectional<br>Case-control study
13	Lemery-Chalfant et al.	2013	*N* (Total) = 582<br>*N* (Twin-pairs) = 291	12 months	Community sample<br>(66% Caucasian; 25% Hispanics; 9% other; US-American; diverse SES)	Cross-sectional
14a	Sadeh et al.	2016	*N* (Total) = 144<br>*N* (SPG) = 93<br>*N* (CG1) = 31<br>*N* (CG2) = 20	0- 24 months<br>(mean SPG= 12.09; mean CG1= 12.40; no significant difference)	Clinical sample and community sample control group (Israeli)	Cross-sectional<br>Case-control study

Note. Papers are listed by publication year and then alphabetically. SPG= sleep problem group; CG= control group; SG= subgroup; SES= socioeconomic status; T1= time one; T2= time two; WS= Williams Syndrome; TD= typically developing.
