# Supplementary material for: Parental Cognitions About Sleep Problems in Infants: A Systematic Review
Source: Front Psychiatry. 2020 Dec 21;11:554221. doi: 10.3389/fpsyt.2020.554221 (PMC7779594; doi:10.3389/fpsyt.2020.554221)
Supplement: Supplementary Table 3 — Child-related cognitions (not referring to sleep)—study sample and design characteristics. [file Table_3.pdf]

Supplemental Table 3: Child-related cognitions (not referring to sleep) - Study sample and design characteristics

study no.	Authors	Publication year	Sample size	Child age at sleep assessment	Sample type and background information	Study design
15	Lozoff, Wolf & Davis	1985	*N* (Total) = 32	6- 48 months	Community sample	Cross-sectional
			N (SPG) = 10	(no significant difference between SPG and CG)	(100% white; US-American)	Control group design
			N (CG) = 22			
16	Scott & Richards	1990	*N* (Total) = 1019	12 months	Community sample	Cross-sectional
			N (SPG) = 266		(English; diverse SES)	Control group design
			N (CG) = 753			
17	Benoit, Zeanah, Parker, Nicholson & Coolbear	1997	*N* (Total) = 37	18- 46 months	Clinical sample and community sample control group (100% Caucasian; Canadian; middle-upper SES)	Cross-sectional
			N (SPG) = 16	(mean= 32.76; no significant difference between SPG and CG)		Case-control study
			N (CG) = 21			
18	Scher & Blumberg	1999	*N* (Total) = 81	12 months	Community sample	Longitudinal
					(Israeli)	Prospective cohort study
19	Touchette et al.	2005	*N* (Total; T1) = 1778	T1: 17 months	Community sample	Cross-sectional
			N (SPG;T1) = 128	T2: 29 months	(88.4% white; Canadian; data from the QLSCD)	Control group design
			N (CG;T1) = 1650			
			N (Total; T2) = 1769			
			N (SPG;T2) = 177			
			N (CG;T2) = 1592			
20	Simard et al.	2008	*N* (Total) = 987	T1: 50 months	Community sample	Longitudinal
				T2: 5 years	(96.7% white; Canadian; data from the QLSCD)	Prospective cohort study
				T3: 6 years		
21		2015	*N* (Total) = 51		Community sample	Cross-sectional

	Millikovsky-Ayalon et al.		*N* (SPG) = 26<br>*N* (CG) = 25	1- 3 years (mean SPG= 1.82; mean CG= 2.16; no significant difference)	(Israeli)	Control group design
22	Zaidman-Zait & Hall	2015	*N* (Total) = 1997	29 months	Community sample<br>(predominantly Caucasian= 93.3%, Canadian; data from the QLSCD)	Longitudinal<br>Prospective cohort study
14b	Sadeh et al.	2016	*N* (Total) = 144<br>*N* (SPG) = 93<br>*N* (CG1) = 31<br>*N* (CG2) = 20	0- 24 months (mean SPG= 12.09; mean CG1= 12.40; no significant difference)	Clinical sample and community sample control group<br>(Israeli)	Cross-sectional<br>Case-control study
24	Tikotzky	2016	*N* (Total) = 80	3- 18 months<br>(mean= 8.8; no significant correlation with outcomes)	Community sample<br>(Israeli; middle-upper SES)	Cross-sectional<br>Correlational design

Note. Papers are listed by publication year and then alphabetically. SPG= sleep problem group; CG= control group; SES= socioeconomic status; T1= time one; T2= time two; T3= time three; QLSCD= Quebec Longitudinal Study of Child Development.
